# Brightening of dark excitons in 2D perovskites

**DOI:** 10.1126/sciadv.abk0904

**Published:** 2021-11-10

**Authors:** Mateusz Dyksik, Herman Duim, Duncan K. Maude, Michal Baranowski, Maria Antonietta Loi, Paulina Plochocka

**Affiliations:** 1 Laboratoire National des Champs Magnétiques Intenses, EMFL, CNRS UPR 3228, University Grenoble Alpes, University Toulouse, University Toulouse 3, INSA-T, Grenoble and Toulouse, France.; 2 Department of Experimental Physics, Faculty of Fundamental Problems of Technology, Wroclaw University of Science and Technology, Wroclaw, Poland.; 3 Zernike Institute for Advanced Materials, University of Groningen, Nijenborgh 4, 9747 AG Groningen, Netherlands.

## Abstract

Optically inactive dark exciton states play an important role in light emission processes in semiconductors because they provide an efficient nonradiative recombination channel. Understanding the exciton fine structure in materials with potential applications in light-emitting devices is therefore critical. Here, we investigate the exciton fine structure in the family of two-dimensional (2D) perovskites (PEA)_2_SnI_4_, (PEA)_2_PbI_4_, and (PEA)_2_PbBr_4_. In-plane magnetic field mixes the bright and dark exciton states, brightening the otherwise optically inactive dark exciton. The bright-dark splitting increases with increasing exciton binding energy. Hot photoluminescence is observed, indicative of a non-Boltzmann distribution of the bright-dark exciton populations. We attribute this to the phonon bottleneck, which results from the weak exciton–acoustic phonon coupling in soft 2D perovskites. Hot photoluminescence is responsible for the strong emission observed in these materials, despite the substantial bright-dark exciton splitting.

## Teaser

Extreme magnetic field reveals the secret of dark states and bright emission of 2D layered perovskites.

## INTRODUCTION

The rapidly emerging field of layered semiconductors provides a timely and exciting opportunity to investigate excitonic physics in the ultimate two-dimensional (2D) limit. In these materials, quantum and dielectric confinement greatly enhances excitonic effects (*1*–*7*), which dominate the optical response even at room temperature. In addition, more subtle effects such as the exciton fine structure are enhanced, facilitating their observation. The exciton fine structure, which results from the exchange interaction between the electron and hole spins (*8*–*11*), systematically induces a splitting of the bright and dark states (*10*–*12*). This splitting can have catastrophic consequences for light emitters that rely on exciton recombination, because the lowest excitonic state is typically dark (*13*–*15*). This is particularly relevant for 2D perovskites that are promising candidates for cheap and efficient light emitters (*16*–*20*).

2D metal halide layered perovskites, consisting of thin slabs of lead or tin halide octahedra separated by large organic molecule spacers, are ideal quantum wells, where excitons are confined in the central metal halide slab, with the organic spacers acting as barriers (*2*, *3*). The bandgap can be tuned by both chemical composition and thickness of the inorganic slab (*21*). In addition, the spacer, which controls dielectric environment and imposes the distortion angles of the octahedral units, can be chosen from a plethora of organic molecules, tuning critical parameters such as the bandgap, carrier effective mass, exciton-phonon coupling, and the excitonic properties (*22*–*30*).

Despite numerous optical investigations, the detailed exciton structure of 2D perovskites remains to be elucidated. The reported values (1 to 30 meV) of the bright-dark exciton splitting vary extensively (*11*, *31*–*34*). Photoluminescence (PL), which is affected by trap states and complex exciton dynamics, provides only a rough estimate of the splitting. In perovskites, with their particularly soft lattice (*14*, *35*), a direct comparison of the bright-dark splitting with the energy of phonons is crucial to understand the thermal population of the excitonic states that control radiative recombination (*13*–*15*). For the future development of 2D perovskites, it is therefore of paramount importance to understand the exciton fine structure, which can also serve as a benchmark for band structure calculations for this complex material system.

Here, we use a large in-plane magnetic field to mix bright and dark exciton states in the phenethylammonium (PEA)–based family of 2D layered perovskites, providing the first direct measurement of the exciton fine structure splitting in these materials. The magnetic field–induced brightening of the dark exciton state permits the direct observation of the absorption related to this state, allowing us to precisely determine the bright-dark exciton splitting. We observe a direct correlation between the obtained bright-dark splitting and the exciton binding energy. A brightening of the dark state is also observed in PL, with the brightened dark state emission dominating already at low magnetic fields ≃1 T. We find that the excitons are not fully thermalized; their temperature is actually higher than the lattice temperature because of the existence of a phonon bottleneck.

## RESULTS AND DISCUSSION

**Fig. 1.**
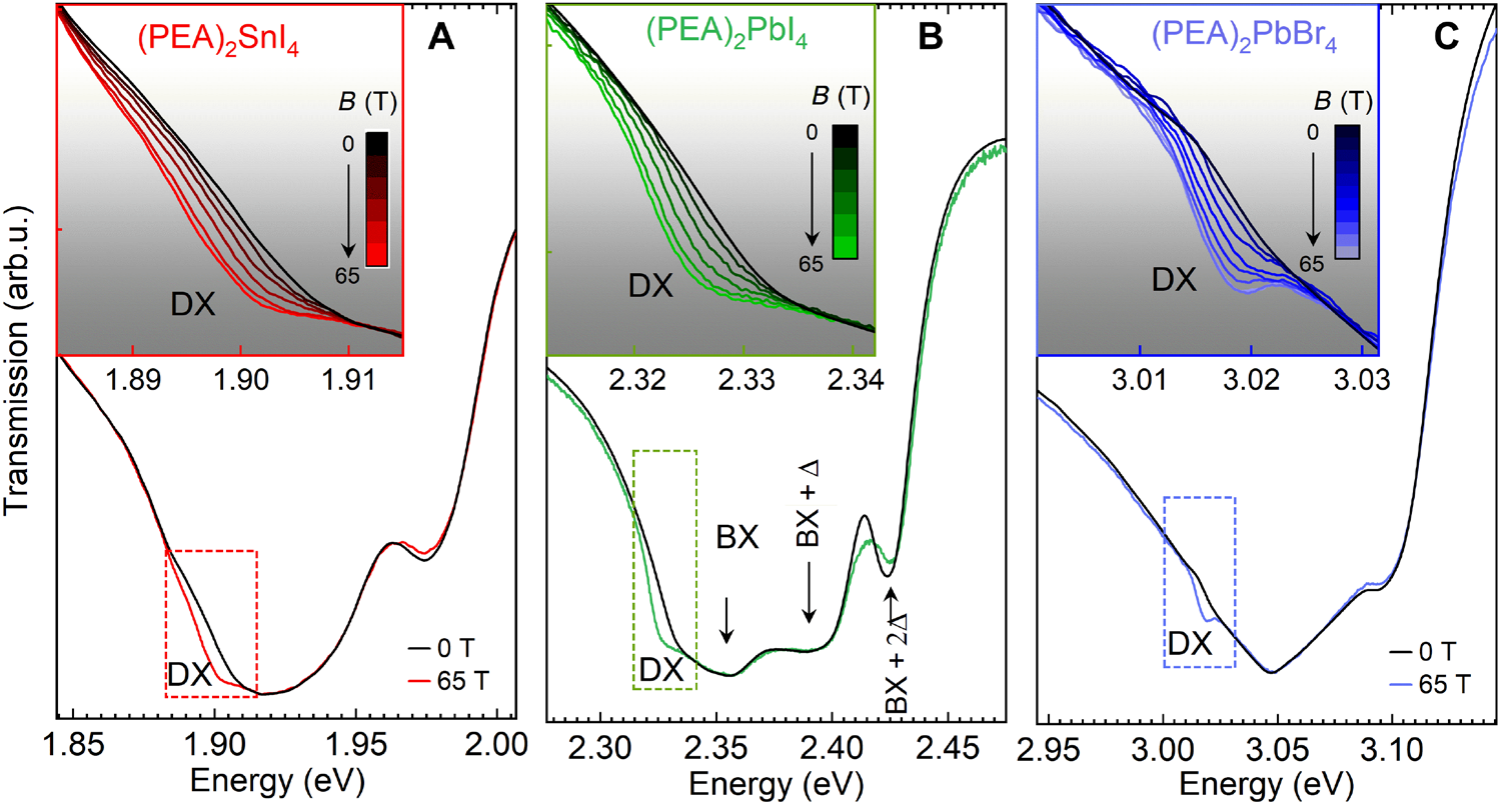
Transmission spectra of 2D perovskites in high magnetic field. (**A** to **C**) Compare spectra measured at zero magnetic field (black) and at *B* = 65 T (colored lines) for (PEA)_2_SnI_4_, (PEA)_2_PbI_4_, and (PEA)_2_PbBr_4_, respectively. The bright exciton (BX) absorption and its phonon replicas (BX + *n*Δ) are resolved for (PEA)_2_PbI_4_. Transmission in the low-energy region indicated by the dashed rectangle shows the appearance of the brightened dark exciton (DX) transition at high magnetic field. Inset in each panel shows an expanded view of the evolution of the dark state with increasing magnetic field, in the spectral range indicated by the dashed rectangle.

Figure 1 (A to C) shows the transmission spectra for (PEA)_2_SnI_4_, (PEA)_2_PbI_4_, and (PEA)_2_PbBr_4_, measured in zero magnetic field and at *B* = 65 T at a temperature of 4.2 K. The magnetic field is applied in the Voigt configuration, i.e., *B* ⊥ **c** and **k** ∥ **c**, where **c** is the stacking direction of the octahedra sheet normal to the substrate and **k** is the light wave vector. The spectra exhibit a characteristic line shape composed of multiple equally spaced absorption minima attributed to the bright free exciton transition (BX) and its phonon replicas (labeled BX + *n*Δ in Fig. 1B) (*27*, *28*).

The application of an external magnetic field notably alters the absorption landscape. In the region highlighted by the dashed rectangle in Fig. 1 (A to C), an additional absorption feature appears, which we identify as the magnetic field brightened (spin-forbidden) dark excitonic state (DX). Transmission spectra, in the spectral range corresponding to the DX feature, are summarized in the insets of Fig. 1 (A to C), where curves for different magnetic fields show a systematic increase of the DX absorption with increasing magnetic field.

**Fig. 2.**
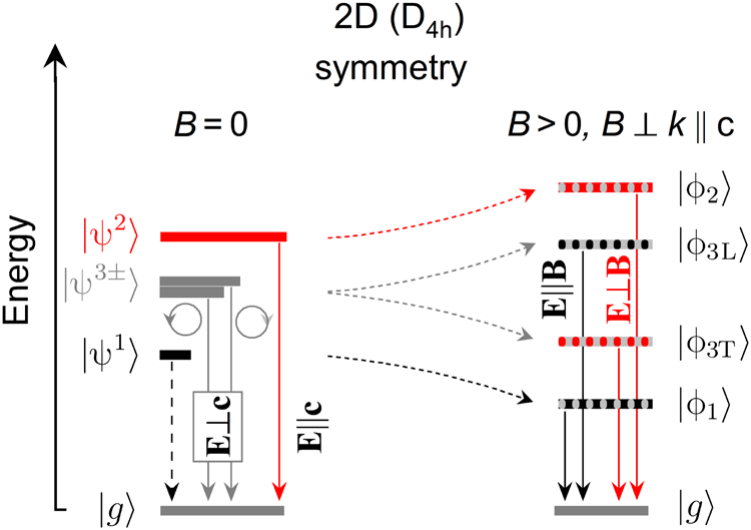
Fine structure splitting of the band-edge excitons for 2D (D_4h_) symmetry. The optical selection rules, allowing to access the respective states, are indicated (**E** is light electric field vector, and **c** is the crystallographic axis perpendicular to quantum well slab). At *B* = 0 T (**left**), ∣*g*〉 is the ground state (no exciton) and ∣ψ^1^〉 is the dark state. The ∣ψ^2^〉 and ∣ψ^3±^〉 states are optically active (bright states) with out-of-plane and in-plane symmetries, respectively. At *B* > 0 and **B** ⊥ **k** ∥ **c** (**right**), all four states (∣ϕ_1_〉, ∣ϕ_2_〉, ∣ϕ_3L_〉, and ∣ϕ_3T_〉) have nonzero dipole moment in the plane of 2D perovskite.

The energy structure of the band-edge excitons is qualitatively the same in 2D and 3D metal halide perovskites (*10*, *11*). In the unperturbed system (*B* = 0), four band-edge excitonic states are presented schematically in Fig. 2: the dark state (ψ^1^), the excitonic state with out-of-plane dipole moment orientation (ψ^2^; usually referred to as a gray exciton), and two excitonic states with in-plane dipole orientation (ψ^3−^ and ψ^3+^), which couple to left- and right-handed circularly polarized light. These excitonic states are built from s-like hole states and p-like electron states, each of them having total angular momentum *J*_e/h_ = 1/2. ψ^1^ (*J* = 0) is a spin-forbidden dark exciton state, and the remaining three (ψ^2^ and ψ^3±^) are optically bright (*J* = 1) with different optical selection rules as indicated in Fig. 2. In particular, the observation of ψ^2^ in absorption-like experiment requires a nonzero electric field (of the probing light) in the out-of-plane direction. Regardless of the underlying structure symmetry, the exchange interaction between electron and hole spins lifts the degeneracy between bright and dark states. In lower symmetry structures, the exchange interaction, together with crystal field, further lifts the degeneracy of bright states (*10*, *11*, *36*, *37*). In 2D perovskites, the broken symmetry in the crystallographic **c** direction lifts the degeneracy of bright exciton states with in-plane (ψ^3+^, ψ^3−^) and out-of-plane dipole moments (ψ^2^). The expected exciton energy ladder is shown in Fig. 2. Here, we would like to emphasize two important aspects related to the exciton ladder: (i) The sign of the crystal field splitting, which determines the energetic position of ψ^2^ with respect to the ψ^3±^ states, is a subject of debate (*38*). Our experimental data suggest that ψ^2^ is pushed to higher energies compared to ψ^3±^, indicating a negative crystal field splitting; (ii) recent studies suggest that the degeneracy of the in-plane excitonic states is lifted in (PEA)_2_PbI_4_ (*39*). However, both of these aspects cannot be tentatively confirmed in the case of our samples because of the large spectral broadening and limited spectral resolution.

In magnetic field, the intrinsic symmetry of the crystal is broken. Therefore, the excitonic states are no longer eigenstates of the Hamiltonian without magnetic field; however, the new eigenstates can still be expressed as a linear combination of exciton states without magnetic field. In the Voigt configuration, the magnetic field mixes the in-plane excitonic states with the dark and out-of-plane excitonic states (*12*, *31*) (in contrast to the Faraday configuration, the ψ_1_ and ψ_2_ states are not mixed with each other). New exciton states form two pairs of states. One pair (ϕ_1_ and ϕ_3L_) is characterized by a dipole moment along *B* and is called longitudinal, while a second pair (ϕ_2_ and ϕ_3T_) is labeled as transverse and has its dipole moment perpendicular to *B*

$$\phi_{1,3L}=c_{1,3L}\psi^{1}+d_{1,3L}(\psi^{3+}-\psi^{3-})$$ (1)

$$\phi_{2,3T}=c_{2,3T}\psi^{2}+d_{2,3T}(\psi^{3+}+\psi^{3-})$$ (2)

Here, we used shortened notation for pairs of states (ϕ_1_, ϕ_3L_) and (ϕ_2_, ϕ_3T_), as their form does not change and differs only with coefficients defined below (the separated formulas for each state can be found in the Supplementary Materials). The formation of two pairs of orthogonal states in magnetic field is schematically depicted in Fig. 2. The energy of the exciton states as a function of magnetic field is described by

$$E_{1,3L}=\frac{1}{2}(E_{1}+E_{3}\pm \sqrt{{\left(E_{1}-E_{3}\right)}^{2}+{\left(g_{L}\mu_{B}B\right)}^{2}})$$ (3)

$$E_{2,3T}=\frac{1}{2}(E_{2}+E_{3}\pm \sqrt{{\left(E_{2}-E_{3}\right)}^{2}+{\left(g_{T}\mu_{B}B\right)}^{2}})$$ (4)

where *g*_L_ = *g*_e⊥_ − *g*_h⊥_ and *g*_T_ = *g*_e⊥_ + *g*_h⊥_ are the effective Landé *g* factors for the longitudinal and transverse states, *g*_e⊥_ and *g*_h⊥_ are the electron and hole *g* factors perpendicular to the **c** axis, following the notation in (*12*), and μ_B_ is the Bohr magneton. The coefficients *c*_1,3L_, *c*_2,3T_, *d*_1,3L_, and *d*_2,3T_, which are all functions of the magnetic field, the splitting of the exciton states, and the effective *g* factors, are given as follows

$$c_{1,3L}=\frac{\frac{1}{\sqrt{2}}g_{L}\mu_{B}B}{\sqrt{2{\left(E_{1,3L}(B)-E_{1}\right)}^{2}+\frac{1}{2}{\left(g_{L}\mu_{B}B\right)}^{2}}}$$ (5)

$$d_{1,3L}=\frac{E_{1}-E_{1,3L}(B)}{\sqrt{2{\left(E_{1,3L}(B)-E_{1}\right)}^{2}+\frac{1}{2}{\left(g_{L}\mu_{B}B\right)}^{2}}}$$ (6)

$$c_{2,3T}=\frac{\frac{1}{\sqrt{2}}g_{T}\mu_{B}B}{\sqrt{2{\left(E_{2,3T}(B)-E_{2}\right)}^{2}+\frac{1}{2}{\left(g_{T}\mu_{B}B\right)}^{2}}}$$ (7)

$$d_{2,3T}=\frac{E_{2,3T}(B)-E_{2}}{\sqrt{2{\left(E_{2,3T}(B)-E_{2}\right)}^{2}+\frac{1}{2}{\left(g_{T}\mu_{B}B\right)}^{2}}}$$ (8)

The magnetic field transfers oscillator strength from the in-plane excitonic states (ψ^3±^) to the dark states, brightening these nominally inaccessible states. At the same time, the absorption related to the bright states is weakened. According to the above equations, the brightened dark state should couple to light with the electric field component along the magnetic field (*E* ∥ *B*; Fig. 2).

**Fig. 3.**
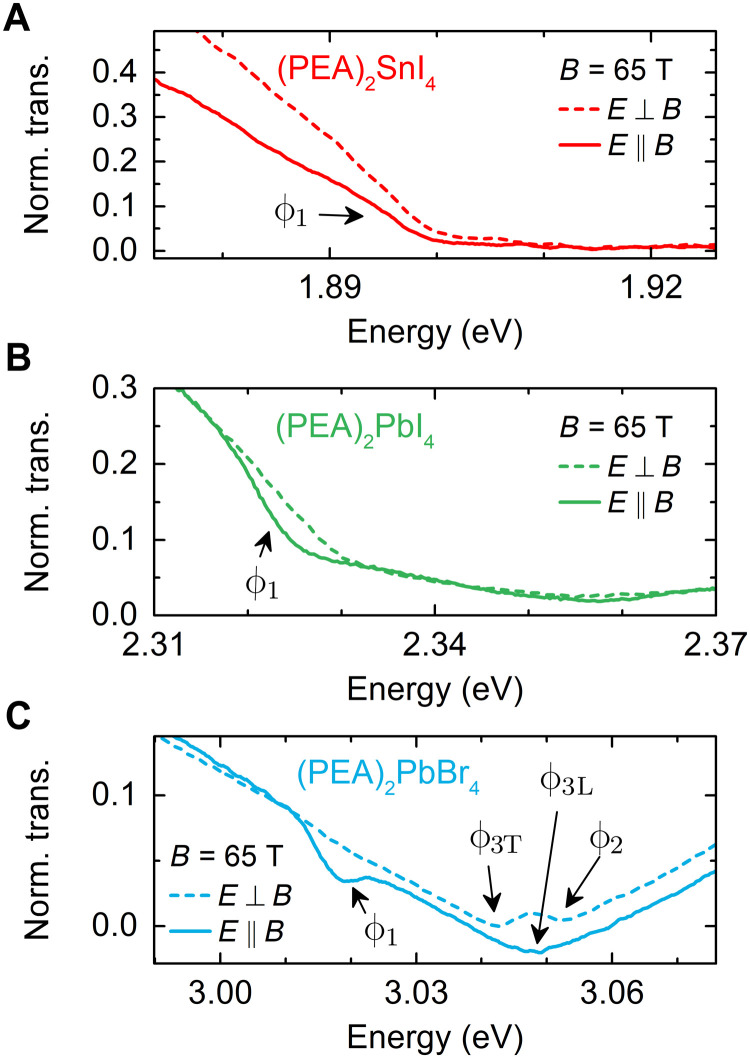
Excitonic states in *B ⊥ E* and *B∥E* polarizations. (**A** to **C**) Transmission spectra for the three compounds measured at a magnetic field of *B* = 65 T, with the excitation light polarized along and normal to the magnetic field *B*.

Transmission measurements in the linear polarization basis validate the optical selection rules. Figure 3 (A to C) compares the transmission spectra measured at *B* = 65 T for two different light polarization, along (*E* ∥ *B*) and normal (*E* ⊥ *B*) to the magnetic field. The DX absorption feature (labeled ϕ_1_) appears only for the *E* ∥ *B* configuration, corroborating the dark state origin of this optical transition.

**Fig. 4.**
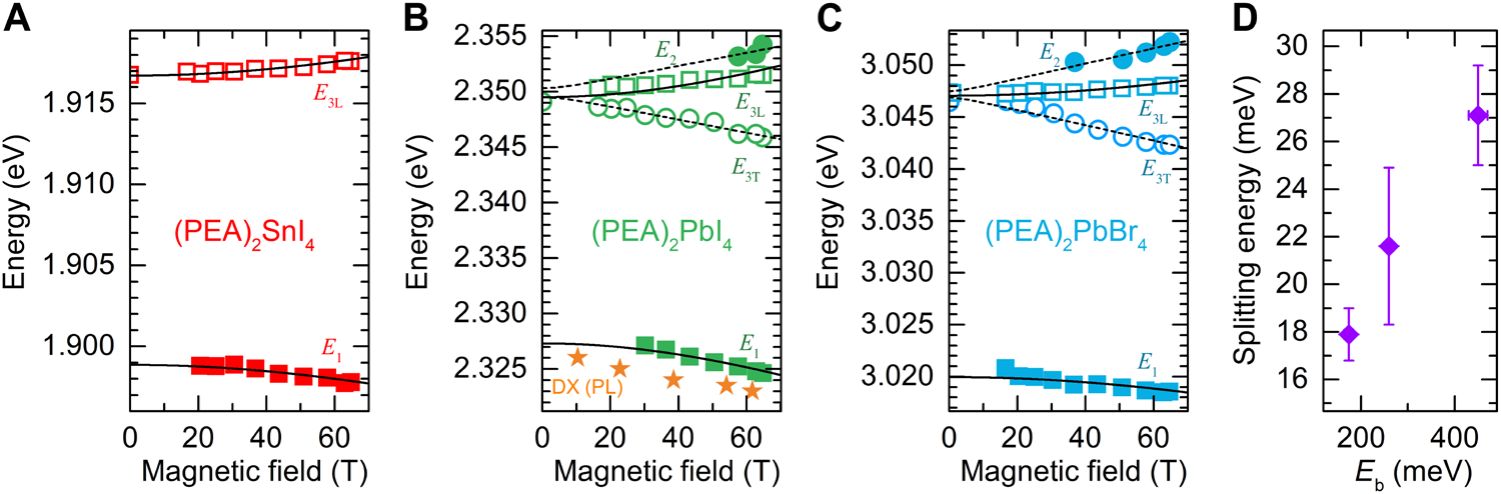
Bright-dark energy splitting in 2D perovskites. (**A** to **C**) Evolution of transition energy for DX and BX states in a magnetic field for the three compounds. The PL emission energy of the brightened dark state for (PEA)_2_PbI_4_ is Stokes-shifted by ≃2.5 meV and follows a similar trend as the DX absorption. Solid and dashed lines are fitted using the expression for *E*_1,3L_ and *E*_2,3T_ given in the text. For (PEA)_2_SnI_4_, the ϕ_2_ state is not resolved (fig. S1). (**D**) Bright-dark energy splitting at *B* = 0 T in function of exciton binding energy *E*_b_ [*E*_b_ is taken after (*23*, *24*) for (PEA)_2_SnI_4_ and (PEA)_2_PbI_4_ and after (*40*) for (PEA)_2_PbBr_4_].

A detailed analysis of the transmission spectra in magnetic field (see also figs. S1 to S3) provides further insight into the exciton fine structure. The magnetic field mixes the bright and dark states into four new eigenstates ϕ_1_, ϕ_3L_, ϕ_2_, and ϕ_3T_, which are clearly visible in the polarization-resolved transmission spectra of (PEA)_2_PbBr_4_ (Fig. 3C) and (PEA)_2_PbI_4_ (fig. S2). Figure 4 (A to C) shows evolution of these states in a magnetic field. The ϕ_2_ state appears on the high energy side of bright states and blue shifts with increasing magnetic field, while ϕ_3T_ red shifts. This allows us to conclude that the order of exciton states in the magnetic field for (PEA)_2_PbI_4_ and (PEA)_2_PbBr_4_ corresponds to the energy ladder presented in Fig. 2. Unfortunately, we are unable to conclude this for the case of (PEA)_2_SnI_4_, because we could not identify the ϕ_2_ state in the spectra (see also fig. S1). This is partially due to the lower exciton binding energy that generally results in smaller fine structure splitting and also due to the typical lower quality of Sn-based samples.

Fitting the experimental data with the expression for *E*_1,3L_(*B*) and *E*_2,3T_(*B*) (curves in Fig. 4, A to C) allows us to extract the bright-dark splitting and the effective Landé *g* factors. The results are summarized in Table 1.

**Table 1.** Summary of measured bright-dark splittings (Δ) and effective *g* factors for longitudinal (*g*_L_* = g*_e⊥_ − *g*_h⊥_) and transverse (*g*_T_* = g*_e⊥_ *+ g*_h⊥_) states in Voigt geometry. Where possible, we calculate the individual electron and hole *g* factors. For completeness, we show the bright-state *g* factors (*g*_e∥_ + *g*_h∥_) measured in Faraday geometry from our previous work (*23*) and new data for (PEA)_2_PbBr_4_ presented in fig. S6.

	**Δ (meV)**	***g*_L_**	***g*_T_**	***g*_e⊥_**	***g*_h⊥_**	***g*_e∥_ *+g*_h∥_**
(PEA)_2_SnI_4_	17.9 ± 1.1	2.3 ± 0.1	–	–	–	1.6 ± 0.1
(PEA)_2_PbI_4_	21.6 ± 3.3	4.0 ± 0.3	1.9 ± 0.5	2.9 ± 0.6	−1.1 ± 0.6	1.2 ± 0.1
(PEA)_2_PbBr_4_	27.1 ± 2.1	3.2 ± 0.3	2.6 ± 0.2	2.9 ± 0.4	−0.3 ± 0.4	0.8 ± 0.1

Figure 4D shows the obtained bright-dark splitting as a function of the exciton binding energy [taken after (*23*, *24*, *40*)]. Extracted bright-dark splitting are about two times higher than recent estimations from temperature-dependent PL studies of (PEA)_2_PbI_4_ or (PEA)_2_SnI_4_ (*32*, *34*). The difference can stem from the fact that PL probes the lowest energy states, while absorption probes the highest density of states. The bright-dark splitting systematically increases with increasing exciton binding energy, in agreement with the general expectations for excitonic complexes (*8*). The measured splitting is an order of magnitude larger than in 3D perovskite nanocrystals (*14*), again in agreement with the enhanced excitonic effects in 2D perovskites.

The values of the effective *g* factors for longitudinal transitions (*g*_L_) are close to the calculated value of 2.65 for MAPbI_3_ (*12*). The similar *g* factors for 2D (PEA)_2_PbI_4_ and 3D MAPbI_3_ are consistent with the similar exciton reduced effective mass for these compounds (*23*, *24*). Because both the effective mass and *g* factor depend on the contribution of atomic orbitals to the band-edge states (*12*), our observations indicate that the band structure in the 2D limit is similar to that of the bulk material.

For completeness, in Table 1, we summarize the effective and individual *g*_⊥_ factors normal to the **c** axis, together with our previously reported data on effective *g* factors along the **c** axis (*g*_e∥_ + *g*_h∥_, Faraday geometry) for the lead and tin variants. To complete the picture in fig. S6, we present unpublished data for (PEA)_2_PbBr_4_ measured in Faraday geometry. From the Zeeman splitting, we estimate the *g*_e∥_ + *g*_h‖_ to be 0.8 ± 0.1. The effective *g* factor (*g*_e_ + *g*_h_) increases when the sample is rotated from the Faraday (*B* ∥ **c**) to the Voigt geometry (*B* ⊥ **c**), which is consistent with theoretical expectations (*12*).

**Fig. 5.**
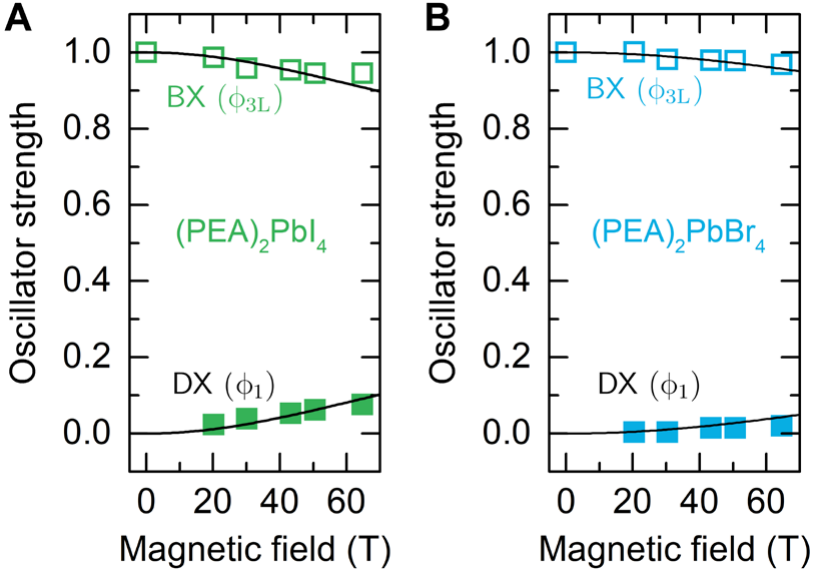
Transition oscillator strength in high magnetic field. (**A** and **B**) Symbols show the oscillator strength for BX and DX states defined as the integrated intensity of each transition normalized by the integrated intensity of the BX transition at *B* = 0 T. Black lines are the calculated oscillator strength as described in the text.

From the transmission data measured in magnetic field, we estimate the change of the oscillator strength of the bright and dark states (figs. S4 and S5). The measured changes in oscillator strength are in good agreement with theory as shown in Fig. 5 (A and B). Because the oscillator strength analysis was performed for spectra taken with *E* ∥ *B* polarization, the enhancement of the brightened dark state ϕ_1_ absorption is accompanied by the weakening absorption of the coupled ϕ_3L_ bright state. The solid lines in Fig. 5 (A and B) correspond to the $d_{1,3L}^{2}$ coefficients describing the contribution of the bright states (ψ^3±^ at *B* = 0 T) to the eigenstates formed by the magnetic field (ϕ_1,3L_ for *B* > 0). The oscillator strengths in Fig. 5 (A and B) are calculated using the previously determined bright-dark splitting and effective *g* factors summarized in Table 1.

**Fig. 6.**
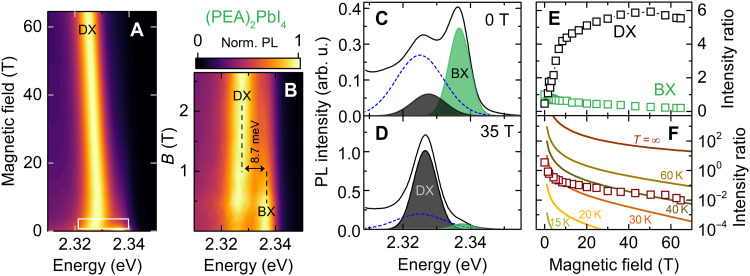
Brightening of dark state observed in magneto-photoluminescence. (**A**) The evolution of PL spectra in the magnetic field for (PEA)_2_PbI_4_ presented in the form of a false-color map. (**B**) Low magnetic field region indicated by a white rectangle in (A). (**C** and **D**) Fitting results to the 0- and 35-T PL spectra, respectively. Dashed lines represent the localized exciton emission dominating over the DX at *B* = 0 T (fig. S7). (**E**) Increase of the BX and DX intensities with respect to the BX intensity at 0 T. (**F**) BX/DX intensity ratio. Solid lines are calculated using Eq. 9 for selected temperatures.

A brightening of the dark state is also observed in PL. In Fig. 6A, we show the PL spectra for (PEA)_2_PbI_4_ [for (PEA)_2_SnI_4_, see fig. S8] as a function of the in-plane magnetic field (Voigt geometry) in the form of a false-color map. For magnetic fields *B* ≥ 3 T, the PL emission is already dominated by the brightened dark exciton recombination. The PL emission shifts toward lower energies at approximately the same rate as the brightened dark exciton absorption (Fig. 4B). At zero magnetic field, the bright excitonic emission is accompanied by a satellite peak on the low energy side (Fig. 6C) with a separation between the peaks of ≃11.2 meV. The low-energy emission is attributed to an exciton localized on trap states, and its contribution to the PL response (dashed line in Fig. 6C) surpasses the contribution of the brightened dark states. With increasing magnetic field, the dark exciton quickly gains in intensity and dominates the PL response. Evidently, the bright-dark state splitting observed in PL is smaller than extracted from absorption studies and equals to 8.7 meV at *B* ≃ 1 T (Fig. 6B). This discrepancy can be understood as an effect of local potential fluctuations. PL emission probes the lowest-lying state, while absorption probes states with the highest density; thus, a Stokes shift of PL with respect to the absorption spectra is typically observed.

In principle, the dark state should not be visible without the magnetic field in the PL emission spectra; however, the selection rules can be relaxed in the presence of crystal distortion, higher-order transition moment processes, and phonon-assisted transitions (*11*). This, together with a much higher occupation probability of dark states compared to bright states at low temperature, results in a detectable emission from the dark state even at *B* = 0 T.

The brightened dark exciton dominates the PL response already at *B* ≃ 3 T (Fig. 6B). Further increase of the magnetic field makes the bright exciton emission barely visible (Fig. 6D). We observe a sixfold increase of the DX PL intensity in magnetic field with respect to the BX intensity at *B* = 0 T (Fig. 6E). The PL intensity is the product of the occupation and the oscillator strength. Therefore, at low temperatures, a small increase of the oscillator strength results in a notable enhancement of the PL intensity of the highly populated dark state. The symbols in Fig. 6F show the intensity ratio of the bright/brightened dark exciton PL emission. Under thermal equilibrium, the intensity ratio can be approximated by the following expression (which neglects ψ_2_)

$$r\approx \frac{{|d_{3L}(B)|}^{2}+{|d_{3T}(B)|}^{2}}{{|d_{1}(B)|}^{2}}\text{exp}\;(-\frac{E_{3}-E_{1}}{k_{B}T})$$ (9)

The solid lines in Fig. 6F are the calculated intensity ratio for selected temperatures. The experimental data points are situated between curves for 30 to 40 K, whereas the measurement was performed at 2.2 K. This suggests that the excitons recombine before they have time to fully thermalize. Hot PL emission is expected in materials with extremely short radiative (PL) lifetimes (*41*). However, in 2D perovskites with radiative lifetimes of at least a few tens of picoseconds at ∼10 K (*34*), one would expect the occupation of the states to follow a Boltzmann distribution.

Nevertheless, a non-Boltzmann distribution can be understood when the particular exciton-phonon coupling in perovskite materials is taken into account. In these soft materials, the coupling of excitons to acoustic phonons is weak in both 3D (*14*) and 2D (*42*, *43*) perovskites. Moreover, because of momentum-energy conservation, the energy change in a single scattering process is in the range of 0.1 meV (*44*). Therefore, acoustic phonons are unable to efficiently scatter excitons between the bright and dark states. Moreover, the bright-dark splitting corresponds to a gap in the density of states of the longitudinal optical (LO) phonon modes that strongly couple to excitonic states. For example, in (PEA)_2_PbI_4_, the LO phonon energies related to inorganic cage and organic spacer are ≃12 to 15 meV and ≃30 to 40 meV, respectively (*27*, *28*). Thus, the energy mismatch slows the relaxation to the dark state, resulting in a higher exciton temperature compared to the lattice temperature (see also extended discussion in the Supplementary Materials). A similar effect is also observed in perovskite nanocrystals, which exhibited strong PL emission at cryogenic temperatures, despite the dark state of the system being the lowest-lying excitonic state (*14*).

In conclusion, using high magnetic fields, we were able to observe the signature of the dark exciton state in the transmission spectra of the representative 2D perovskite (PEA)_2_SnI_4_, (PEA)_2_PbI_4_, and (PEA)_2_PbBr_4_. In agreement with the general expectation for semiconductors, our investigation shows that the lowest-lying exciton state is dark in all cases. In-plane magnetic field brightens the dark excitonic states, allowing them to be directly observed in absorption spectra. The optical selection rules, derived from symmetry consideration, are confirmed by the (brightened) dark state absorption, which is observed only when the electric field of the light is parallel to the magnetic field. The extracted bright-dark splittings are in the range of 18 to 30 meV at *B* = 0 T. The brightening is also observed in PL, with the brightened dark exciton emission dominating already at *B* ≃ 3 T. The evolution of the PL signal in the magnetic field suggests that, at low temperatures, the exciton population is not fully thermalized because of the existence of a phonon bottleneck, which occurs due to the specific nature of the exciton-phonon coupling in soft perovskite materials.

## MATERIALS AND METHODS

### Optical spectroscopy setup

Transmission spectra were measured in a nitrogen-cooled pulsed magnet, providing a maximum field of *B* ≃ 65 T with a pulse duration of ≃500 ms. A tungsten halogen lamp was used as a broadband white light source. The measurements were performed in the Voigt configuration, with the **c** axis of the sample perpendicular to the magnetic field and parallel to the **k** vector of the light. White light is sent to the sample using an optical fiber, and transmitted light is collected using a lens coupled to a second fiber. The transmitted light is dispersed and detected using a monochromator equipped with a diffraction grating and a nitrogen-cooled charge-coupled device camera. The sample is installed in a helium cryostat in the center of the magnetic field. Unless otherwise stated, all data were taken with the sample in pumped liquid helium at *T* = 2.2 K. PL spectra are acquired in the same geometry, and linear polarization was resolved in situ using a broadband polarizer.

### Sample synthesis details

Glass substrates were ultrasonically cleaned sequentially using detergent solution, deionized water, acetone, and isopropanol. Subsequently, the substrates were dried in an oven *T* = 140°C for ≥10 min before treatment with ultraviolet ozone for 20 min. Immediately after cleaning, the substrates were placed in a nitrogen-filled glove box for film deposition.

A stoichiometric precursor solution was used, prepared by dissolving PEA (Phenethylammonium iodide, 98.0% TCI) and PbI_2_ or SnI_2_ at a molar ratio of 2:1 in a mixed solvent of *N*,*N*′-dimethylformamide and dimethyl sulfoxide (4:1 volume ratio, 0.5 M concentration). To homogenize the solutions, they were stirred for at least 3 hours at room temperature before deposition. A spin-coating process with antisolvent treatment was used to deposit the precursor solution onto the cleaned substrates. A rotation speed of 2000 rpm was used for the first 10 s of the spin-coating process. The speed was then accelerated to 8000 rpm for the remaining 30 s. Five seconds before the end of the spin-coating cycle, the antisolvent (chlorobenzene) was added to the substrate. The films were immediately annealed at 100°C in a nitrogen atmosphere for 10 min.

## Supplementary Materials

### This PDF file includes:

Figs. S1 to S8 Supplementary text and discussion
