# Supplementary material for: Brightening of dark excitons in 2D perovskites
Source: Sci Adv. 2021 Nov 10;7(46):eabk0904. doi: 10.1126/sciadv.abk0904 (PMC8580304; doi:10.1126/sciadv.abk0904)
Supplement: Supplementary file 1 — Figs. S1 to S8 Supplementary text and discussion [file sciadv.abk0904_sm.pdf]

Science Advances
AAAS

Supplementary Materials for

Brightening of dark excitons in 2D perovskites

Mateusz Dyksik, Herman Duim, Duncan K. Maude, Michal Baranowski,
Maria Antonietta Loi, Paulina Plochocka*

*Corresponding author. Email: paulina.plochocka@lncmi.cnrs.fr

Published 10 November 2021, *Sci. Adv.* **7**, eabk0904 (2021)
DOI: 10.1126/sciadv.abk0904

This PDF file includes:

Figs. S1 to S8
Supplementary text and discussion

SUPPLEMENTARY FIGURES

FIG. S1. **Transmission ratio spectra for $(PEA)_2SnI_4$. Energy shift of the BX state.** (a) Transmission ratio spectra T(B)/T(0) showing the influence of the magnetic field on the transmission spectra in the spectral range of 1s exciton state. At the low energy side at $\simeq 1.9\,\text{eV}$ a strong feature related to the DX is visible. This low energy feature cannot be reproduced by simulating the T_{65T}/T_{0T} spectra by shifting the 0 T spectra by 1 meV i.e. $T_{0T\to 1\,\text{meV}}/T_{0T}$ [22,23,37] thus the low energy signal is a state which gain intensity when the magnetic field rises. (c) The energy difference (at 65 T) between the DX state visible in the T_{65T}/T_{0T} spectra and BX state in the T_{65T} spectra. For reference we plot the transmission spectra at 0 and 65 T spectra in black and red, respectively. (d) Due to the large broadening the energy shift of the BX state is determined from the energy shift of the 2nd phonon replica [23]

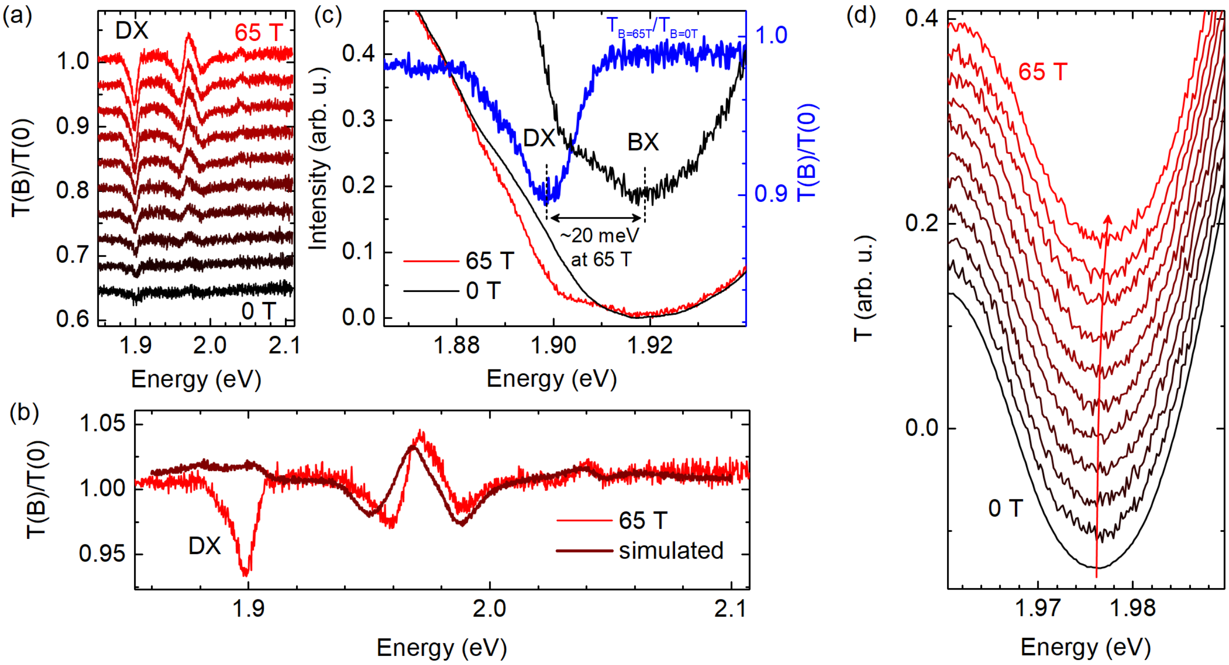

FIG. S2. **Transmission ratio spectra for $(PEA)_2PbI_4$. Signatures of the ϕ_2 state.** (a) Transmission ratio spectra T(B)/T(0) showing the influence of the magnetic field on the transmission spectra in the spectral range of excitonic transitions. At the low energy side at $\simeq 2.33$ eV a strong signal related to the DX is visible. This signal exhibits negative amplitude in the T_B/T_0 spectra, indicating that the new absorbing state is red shifting in magnetic field. On the high energy side of DX we observe a signal with positive amplitude related to ϕ_{3L} and labeled BX (dotted line shows the baseline). (b) The DX signal cannot be reproduced by simulating the T_{65T}/T_{0T} spectra by shifting the 0 T spectra by 1.2 meV i.e. $T_{0T\rightarrow 1.2\,\mathrm{meV}}/T_{0T}$ [22,23,37] thus the low energy signal develops when the magnetic field is on. (c) The 2nd derivative spectra ($|\frac{d^2T}{dE^2}|$) showing the signature of ϕ_2 out-of-plane excitonic state. The signature of this state is also visible on the high energy side of the 2nd phonon replica in panel (d). Here the ϕ_2' is the phonon replica of ϕ_2.

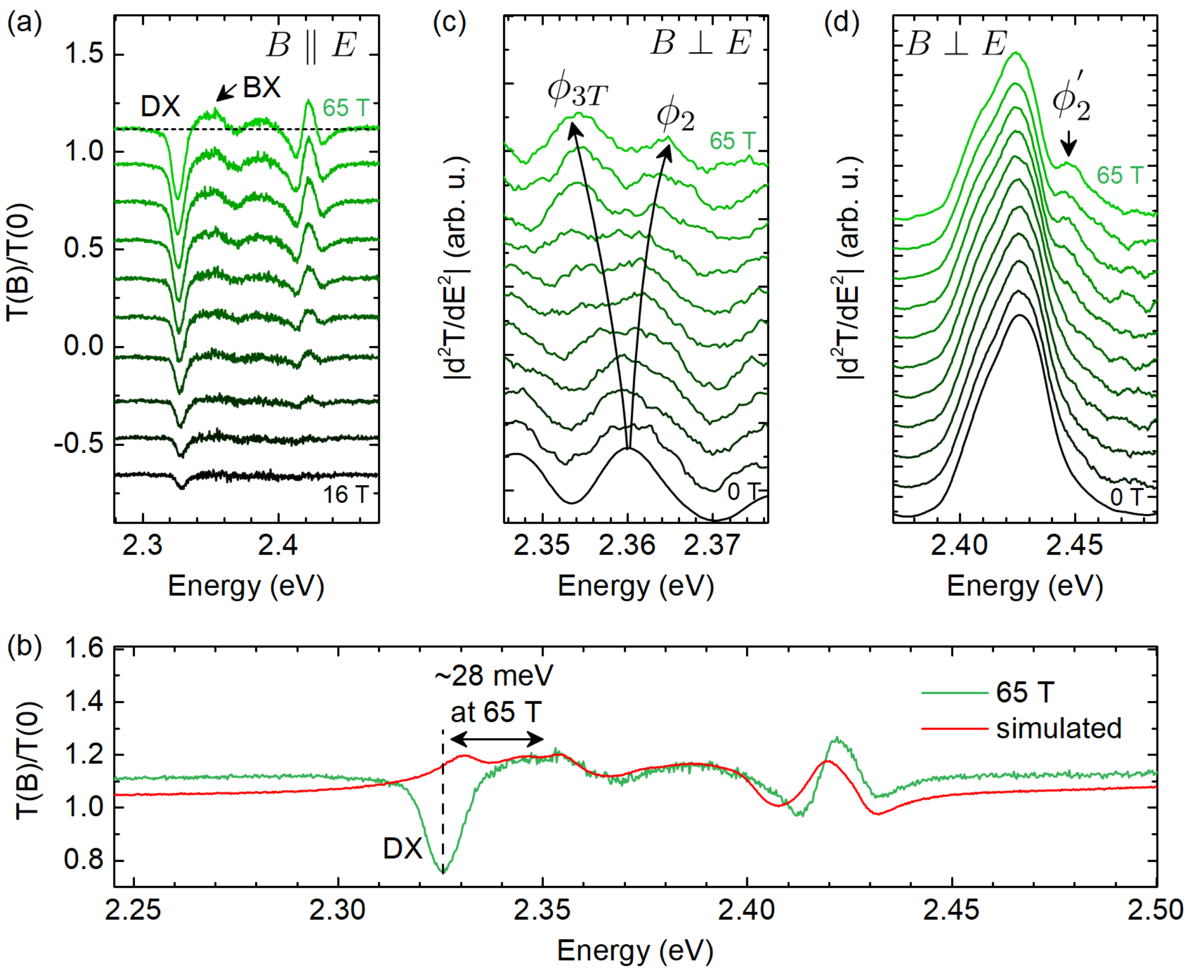

FIG. S3. **Transmission spectra for $(PEA)_2PbBr_4$.**

Transmission spectra measured for the (a) $B \parallel E$ and (b) $B \perp E$ polarizations. In the high magnetic field (B=65 T, bottom curve) we observed the development of new spectral features related to ϕ_1 and ϕ_2 states.

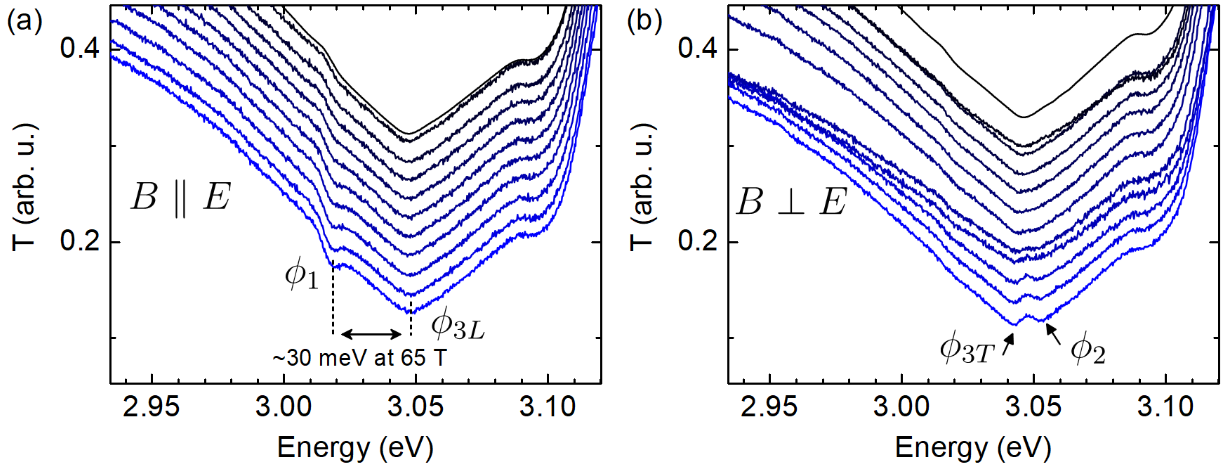

FIG. S4. **Oscillator strength of the dark states in $(PEA)_2PbI_4$.**

In order to reproduce the transmission spectra measured without the magnetic field (black bottom curve) three Voigt-profiled components were used (dashed green) plus an additional component to mitigate the broad background (dashed gray). The cumulative fit (dashed magenta) overlaps well with the measured signal. The oscillator strength of the bright exciton (BX) corresponds to the area under the low-energy component at $\simeq 2.35\,\mathrm{eV}$. For the spectra measured with magnetic field an additional component was used (dot black), corresponding to the increasing absorption due to the dark excitonic state (DX).

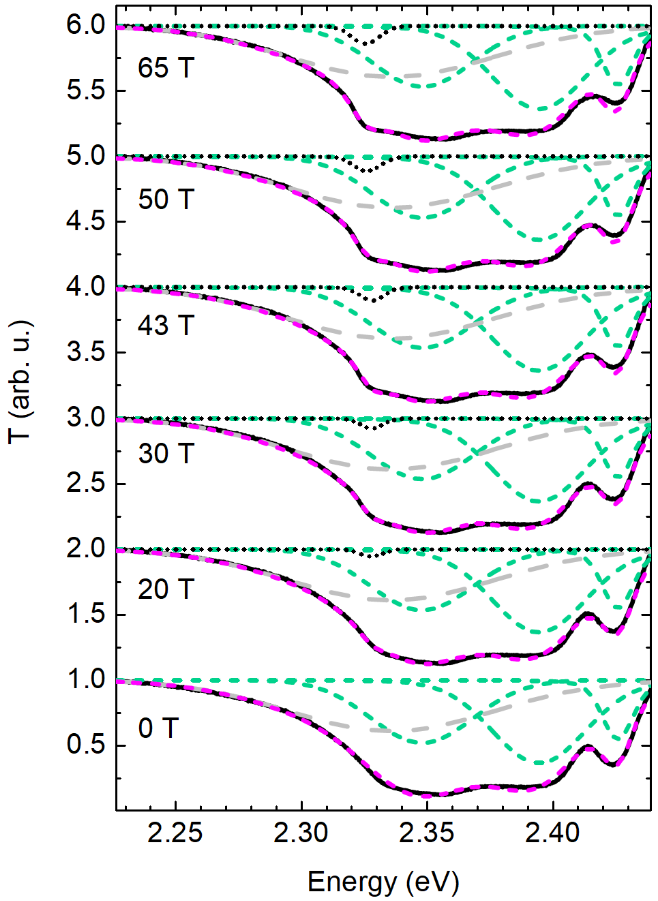

FIG. S5. **Oscillator strength of the dark states in $(PEA)_2PbBr_4$.**

In order to reproduce the transmission spectra measured without the magnetic field (black bottom curve) two Voigt-profiled components were used (dashed blue) plus an additional component to mitigate the broad background (dashed gray). The cumulative fit (dashed magenta) overlaps well with the measured signal. The oscillator strength of the bright exciton (BX) corresponds to the area under the low-energy component at $\simeq 3.05\,\text{eV}$. In the magnetic field an additional component develops (dot black), corresponding to the increasing absorption due to the dark excitonic state (DX).

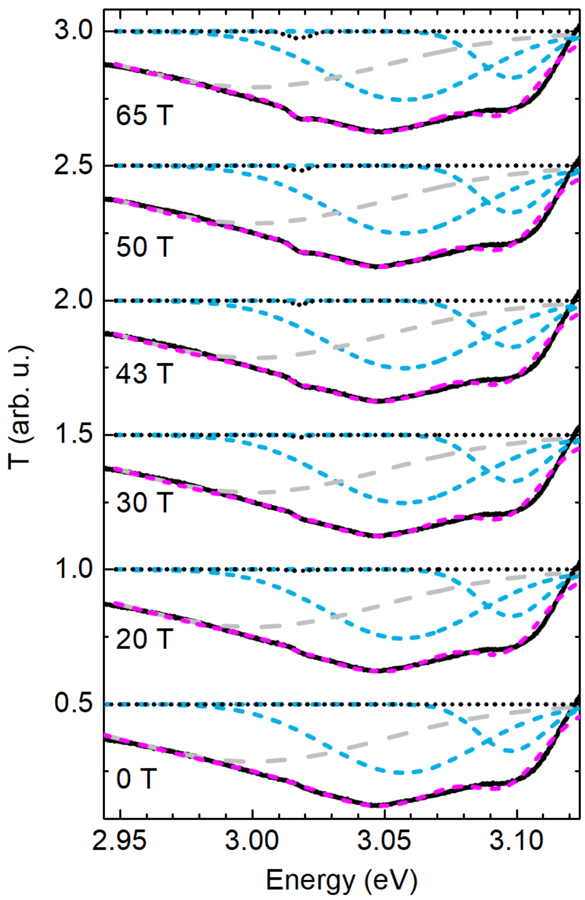

FIG. S6. **Zeeman splitting in $(PEA)_2PbBr_4$.**

(a) Transmission spectra of $(PEA)_2PbBr_4$ measured for two circular polarizations $\sigma+$ and $\sigma-$ for selected strengths of magnetic field. The energy shift of the 1s exciton ($\simeq 3.05\,\mathrm{eV}$) in the function of magnetic field. Solid lines stand for $\Delta E = \pm\frac{1}{2}(g_{e\|} + g_{h\|})\mu_B B + c_0 B^2$, where μ_B is the Bohr magneton. The fitting yields effective g-factor $g_{e\|} + g_{h\|} = 0.8 \pm 0.1$ and diamagnetic coefficient $c_0 = 0.07 \pm 0.01\ \frac{\mu eV}{T^2}$.

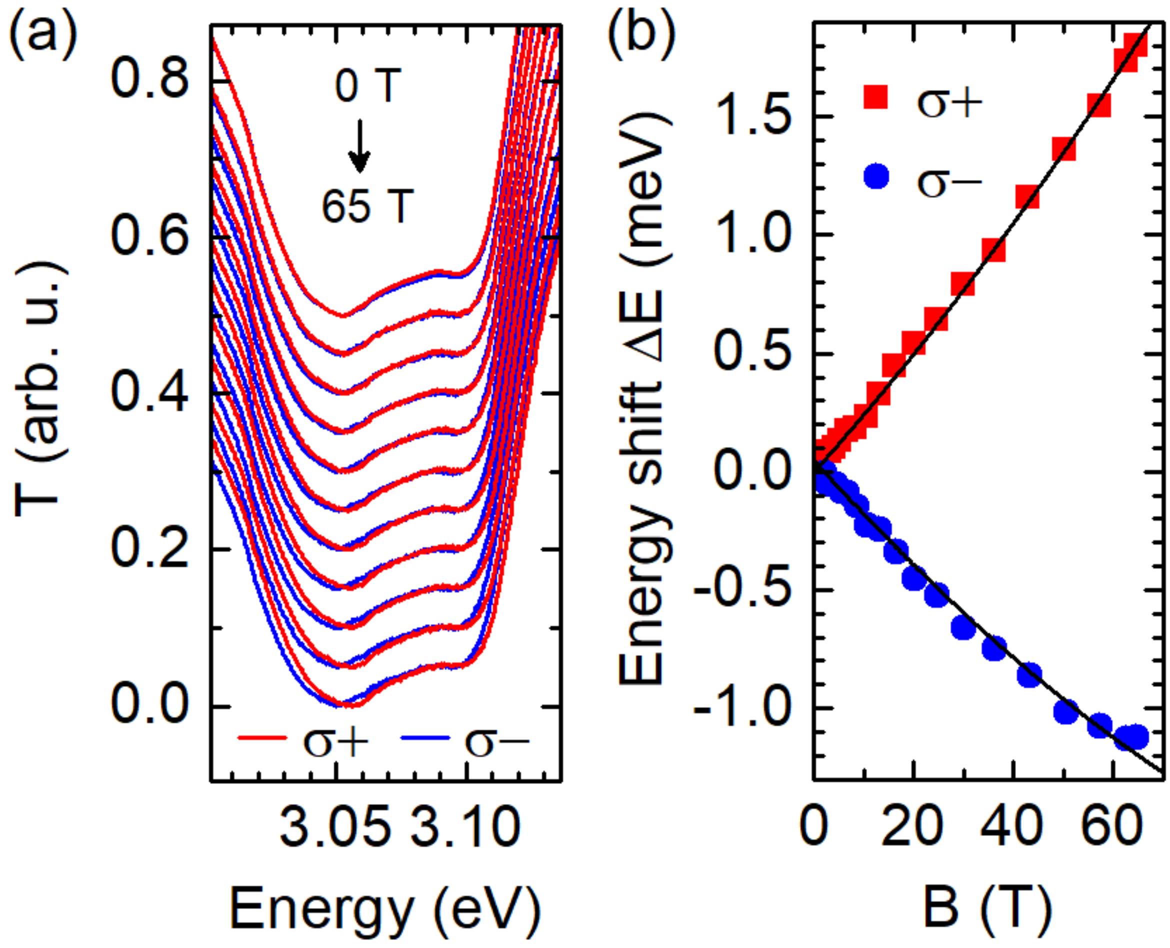

FIG. S7. **PL spectra at low magnetic field.**

(a) PL spectra in the magnetic field $B < 2.6\,\mathrm{T}$. LX = localized exciton. (b) PL energy vs. magnetic field. The DX dominates the PL response already at $B \simeq 1\,\mathrm{T}$ and hinders the LX emission.

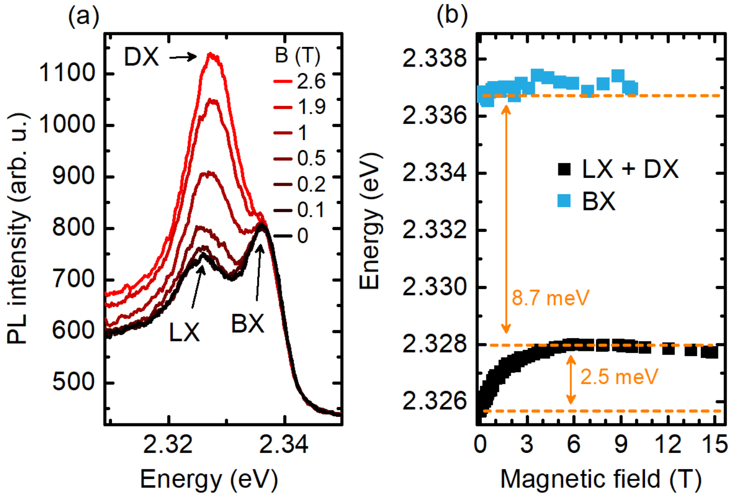

FIG. S8. **PL spectra of $(\mathbf{PEA})_2\mathbf{SnI}_4$ in the magnetic field.**

(a) The evolution of PL spectra in the magnetic field presented in the form of a false-color map. (b,c) Fitting results to the 1 T and 35 T PL spectra, respectively. (d) The increase of the BX and DX intensities with respect to the BX intensity at 0 T. (e) The BX/DX intensity ratio. Solid lines stand for $r \approx \frac{|d_{3L}(B)|^2+|d_{3T}(B)|^2}{|d_1(B)|^2}\exp\left(-\frac{E_3-E_1}{k_B T}\right)$ (see main text) for selected temperatures.

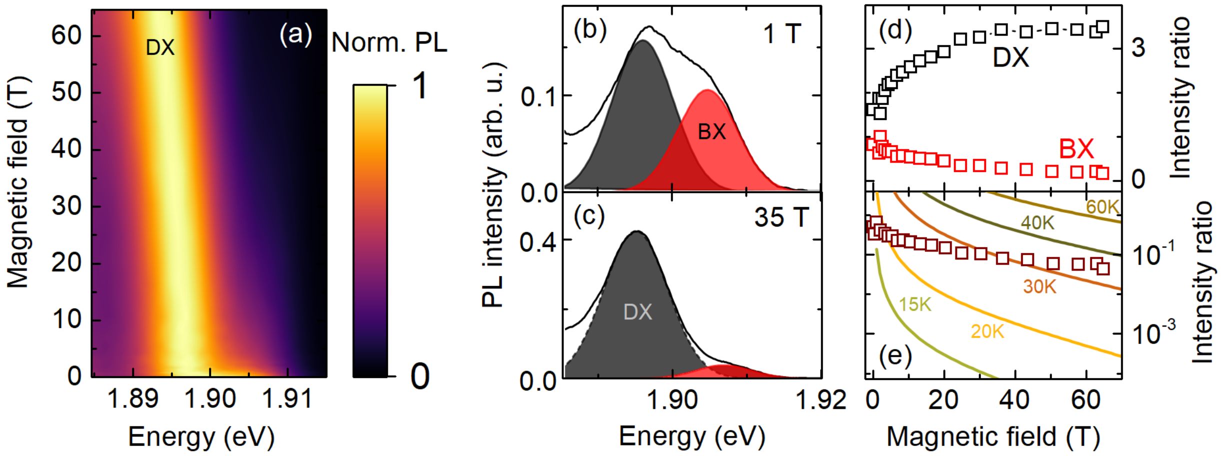

EXCITON STATES IN MAGNETIC FIELD UNDER VOIGT CONFIGURATION.

Under the magnetic field the intrinsic symmetry of the crystal is broken. Therefore the exciton states are no longer eigenstates of the Hamiltonian without magnetic field. The new eigenstates ϕ can be expressed as a linear combination of exciton states without magnetic field ψ. In the Voigt configuration the magnetic field mixes the in-plane excitonic states with the dark and out-of-plane excitonic states [12,31] (in contrast to Faraday configuration the stats ψ_1 and ψ_2 are not mixed with each other). New exciton states form two pairs of states. One pair (ϕ_1 and ϕ_{3L}) is characterized by the dipole moment along B and is called longitudinal while second pair (ϕ_2 and ϕ_{3T}) has its dipole moment perpendicular to B. Each of the states at $B \neq 0$ contain bright longitudinal ($\psi^{3+} - \psi^{3-}$) or transverse ($\psi^{3+} + \psi^{3-}$) component and dark ψ^1 or out-of-plane component ψ^2:

$$\phi_1 = c_1\psi^1 + d_1(\psi^{3+} - \psi^{3-}) \tag{1}$$

$$\phi_{3L} = c_{3L}\psi^1 + d_{3L}(\psi^{3+} - \psi^{3-}) \tag{2}$$

$$\phi_2 = c_2\psi^2 + d_2(\psi^{3+} + \psi^{3-}) \tag{3}$$

$$\phi_{3T} = c_{3T}\psi^2 + d_{3T}(\psi^{3+} + \psi^{3-}) \tag{4}$$

The energies of the exciton states as a function of magnetic field are described by (assuming state order as presented in manuscript):

$$E_1(B) = \frac{1}{2}\Big(E_1 + E_3 - \sqrt{(E_1 - E_3)^2 + (g_L\mu_B B)^2}\Big) \tag{5}$$

$$E_{3L}(B) = \frac{1}{2}\Big(E_1 + E_3 + \sqrt{(E_1 - E_3)^2 + (g_L\mu_B B)^2}\Big) \tag{6}$$

$$E_2(B) = \frac{1}{2}\Big(E_2 + E_3 + \sqrt{(E_2 - E_3)^2 + (g_T\mu_B B)^2}\Big) \tag{7}$$

$$E_{3T}(B) = \frac{1}{2}\Big(E_2 + E_3 - \sqrt{(E_2 - E_3)^2 + (g_T\mu_B B)^2}\Big) \tag{8}$$

where $g_L = g_{e\perp} - g_{h\perp}$ and $g_T = g_{e\perp} + g_{h\perp}$ are the effective Landé g-factors for the longitudinal and transverse states, $g_{e\perp}$ and $g_{h\perp}$ are the electron and hole g-factors perpendicular to the c-axis, following the notation in [12], and μ_B is the Bohr magneton.

The coefficients c_1, c_{3L} ,c_2, c_{3T}, d_1, d_{3L}, d_2, d_{3T} which are all functions of the magnetic field, the splitting of the exciton states, and the effective g-factors, are given as follows:

$$c_1 = \frac{\frac{1}{\sqrt{2}} g_L \mu_B B}{\sqrt{2(E_1(B) - E_1)^2 + \frac{1}{2}(g_L \mu_B B)^2}} \tag{9}$$

$$c_{3L} = \frac{\frac{1}{\sqrt{2}} g_L \mu_B B}{\sqrt{2(E_{3L}(B) - E_1)^2 + \frac{1}{2}(g_L \mu_B B)^2}} \tag{10}$$

$$d_1 = \frac{E_1 - E_1(B)}{\sqrt{2(E_1(B) - E_1)^2 + \frac{1}{2}(g_L \mu_B B)^2}}, \tag{11}$$

$$d_{3L} = \frac{E_1 - E_{3L}(B)}{\sqrt{2(E_{3L}(B) - E_1)^2 + \frac{1}{2}(g_L \mu_B B)^2}}, \tag{12}$$

$$c_2 = \frac{\frac{1}{\sqrt{2}} g_T \mu_B B}{\sqrt{2(E_2(B) - E_2)^2 + \frac{1}{2}(g_T \mu_B B)^2}}, \tag{13}$$

$$c_{3T} = \frac{\frac{1}{\sqrt{2}} g_T \mu_B B}{\sqrt{2(E_{3T}(B) - E_2)^2 + \frac{1}{2}(g_T \mu_B B)^2}}, \tag{14}$$

$$d_2 = \frac{E_2(B) - E_2}{\sqrt{2(E_2(B) - E_2)^2 + \frac{1}{2}(g_T \mu_B B)^2}}. \tag{15}$$

$$d_{3T} = \frac{E_{3T}(B) - E_2}{\sqrt{2(E_{3T}(B) - E_2)^2 + \frac{1}{2}(g_T \mu_B B)^2}}. \tag{16}$$

It is easy to notice that when $B \to 0$ then $\phi \to \psi$.

DISCUSSION OF PHONON-BOTTLENECK.

While the detailed analysis and explanation of phonon-bottleneck effect is certainly beyond the scope of our work, based on the textbook description of carrier-phonon scattering in semiconductors we can point out some of the properties of perovskites that can favor the formation of a phonon bottleneck between bright and dark states. Let us first consider the acoustic phonons bottleneck. It is a well established phenomena in perovskite physics observed not only in 3D materials [14] but also for 2D derivatives [42] since perovskites are soft materials. In general, the carriers/excitons weakly couple to acoustic phonons with deformation potential for acoustic phonons D several times lower than for archetypical GaAs

(1.9 eV vs 7.8 eV). Since the electron scattering rate on acoustic phonons is proportional to D^2, we can expect a decrease of the carriers scattering rate on acoustic phonons. This very rough prediction finds support in the temperature dependence of the photoluminescence response, which is particularly well elaborated in [14] for 3D and examples for $(PEA)_2PbI_4$ can be found in [42,43]. Moreover in the context of the bright-dark scattering mechanism we have to be aware that there are important restrictions related to the energy-momentum conservation for acoustic phonon scattering. Namely, the energy-momentum conservation (due to significantly different dispersion of carriers and phonon bands) imposes maximum change of the carrier energy in the range of 0.1 meV for GaAs [44], thus in perovskites with its soft lattice it will be even lower. At the same time the energy separation between the bright and dark states in 2D perovskite is much larger, as we have shown in the range of tens of meV. Therefore, there is an evident energy mismatch between the allowed carrier energy change and initial and final states energy difference in scattering process. We expect that the same applies to optical phonon scattering (both deformation potential and polar scatterings). Obviously in this case there is no restriction related to the momentum conservation (different phonon dispersion), however the problem of the energy mismatch between the bright-dark splitting energy and phonons energy is still valid. For example, the direct scattering from the bright state light cone to brightened dark states light cone is not possible, therefore we observe signs of non-Boltzmann distribution in PL spectra.
